# A Latent Profile Analysis of Vaccine Decision-Making Among Black Adults in South Central Los Angeles

**DOI:** 10.3390/vaccines14080662

**Published:** 2026-07-28

**Authors:** Emilia J. Fields, Cynthia Davis, Francisco Perez, Suellen Hopfer

**Affiliations:** 1 Department of Health, Society & Behavior, UC Irvine Joe C. Wen School of Population & Public Health, University of California, Irvine, CA 92697, USA; shopfer@hs.uci.edu; 2 College of Medicine, Charles R. Drew University of Medicine and Science, Los Angeles, CA 90059, USA; cynthiadavis@cdrewu.edu; 3 College of Science and Health, Charles R. Drew University of Medicine and Science, Los Angeles, CA 90059, USA; 4 Granada on Broadway Outreach Project, Los Angeles, CA 90061, USA; granadaonbroadway@gmail.com

**Keywords:** vaccine acceptance, vaccine equity, health communication

## Abstract

**Background**: Black adults are frequently portrayed as uniformly vaccine-hesitant, obscuring heterogeneity in the attitudes, beliefs, and decision-making processes that shape vaccine decisions. **Methods**: Guided by the socioecological and 5C frameworks, a latent profile analysis was conducted to identify distinct and meaningful profiles of vaccine decision-making among Black adults in South Central Los Angeles, a historically disinvested and medically underserved community (N = 203). **Results**: Three profiles emerged: Empowered and Engaged Trusters (52.0%), Unempowered and Unengaged Noncommitters (32.2%), and Empowered and Engaged Skeptics (15.8%). Subsequent analyses identified healthcare utilization, age, and voting affiliation as predictors of profile membership. Additionally, profile membership was associated with five vaccination outcomes in expected directions, with Trusters reporting higher vaccination intentions and past uptake than both Noncommitters and Skeptics. **Conclusions**: Study findings underscore the heterogeneity in vaccine decision-making among Black adults and offer insight into the within-group diversity in vaccine acceptance beyond hesitancy. Findings have implications for tailored interventions that maintain confidence, overcome apathy, and reduce skepticism to better promote vaccine acceptance and uptake in Black disinvested communities.

## 1. Introduction

Vaccination is a safe and cost-effective public health strategy for reducing incidence, morbidity, and mortality associated with vaccine-preventable diseases. However, across routinely recommended vaccines, coverage remains lower among Black adult populations compared with other racial and ethnic groups in the United States [1]. Black individuals are disproportionately affected by preventable diseases, including COVID-19 and seasonal influenza [2,3]. In historically disinvested Black communities shaped by systemic racism and stifled economic development, social inequities often create conditions that heighten the risk of exposure and poorer health outcomes in ways that can exacerbate and perpetuate existing health inequities [4]. Subsequently, increasing vaccination coverage is urgently needed to improve and maintain health throughout the life course.

Efforts to increase vaccination within Black communities must include dismantling systemic racism, eliminating longstanding inequities, advancing vaccine equity, and promoting vaccine acceptance [5]. Vaccine acceptance refers to an individual’s decision to accept a vaccine when presented with the opportunity to vaccinate. Importantly, this decision reflects an underlying decision-making process shaped by multi-level determinants [6].

Research on vaccine acceptance among Black populations has largely focused on vaccine hesitancy, with studies identifying significant differences in hesitancy between Black and White adults [7,8]. Such findings have contributed to the widespread labeling of Black populations as vaccine-hesitant [9], a term inconsistently operationalized and often conflated with structural inequities in access [10]. This characterization obscures within-group diversity in vaccine acceptance, treating Black adults as a monolithic group despite evidence to the contrary [11,12,13,14]. Notably, there is far less attention to the underlying vaccine decision-making process, contributing to a limited understanding about why vaccine acceptance may vary.

Scholars have called for research to uncover diversity within Black populations [11], and to more broadly examine the interplay of factors that shape vaccine decision-making [15]. The present study addresses research gaps by employing latent profile analysis (LPA). LPA is a person-centered approach that models unobserved heterogeneity and identifies latent subgroups within a given population based on response patterns across multiple variables [16]. Despite its value, LPA remains largely underutilized in the field.

This study conducted an LPA to identify distinct and meaningful profiles of vaccine decision-making among Black adults in South Central Los Angeles, a historically disinvested and medically underserved neighborhood located in Southern California. Guided by the socioecological and 5C frameworks [17,18], the LPA modeled 12 psychological determinants of vaccine decision-making capturing influences across individual, social, institutional, and policy levels: vaccine-related attitudes and beliefs, including vaccine confidence, conspiracy beliefs, risk perceptions, perceived barriers to vaccination; trust in health institutions, mass media, and personal networks for information; decisional engagement, including preference for extensive information seeking and deliberation; and self-efficacy for making informed vaccine decisions. Subsequent analyses examined sociodemographic predictors of profile membership and how past vaccination behaviors and future intentions aligned with the identified profiles. By uncovering the heterogeneity in vaccine decision-making, this study offers insights that informs tailored interventions for promoting vaccine acceptance and uptake within Black communities.

## 2. Materials and Methods

### 2.1. Study Setting and Participants

This study employed a cross-sectional research design. A sample of 203 Black adults were recruited between July 2024 and April 2025 to complete a survey. The survey was available in both print and online formats and was estimated to take approximately 25 min to complete. To reduce response fatigue, the study team orally administered the survey when requested. Participants received a $45 gift card as compensation.

The recruitment strategy was implemented in collaboration with Granada on Broadway Project, a local community-based organization, and Charles R. Drew University of Medicine and Science. This organization offers free health and social services to residents of South Central Los Angeles, primarily serving those who are socially and economically marginalized. Charles R. Drew University partners with this organization to deliver primary healthcare services to residents in non-traditional clinical settings, including COVID-19 vaccinations via mobile clinics during the pandemic. Recruitment employed convenience and snowball sampling, occurring on-site at the local community center, at neighborhood events, and through peer referrals. Eligibility criteria for participation in the study included English-speaking Black adults aged 18–45 residing in South Central Los Angeles. Ethics approval for this study was granted by the institutional review board at the University of California, Irvine (No. 3946).

### 2.2. Study Measures

The unidimensionality of multi-item scales was established using factor analysis. Unless otherwise noted, all multi-item scales used a 5-point response format ranging from 1 (strongly disagree) to 5 (strongly agree). Also, for all multi-item scales, higher mean scores indicated higher endorsement of the corresponding domain.

Psychological antecedents of vaccination. The 15-item 5C scale by Betsch et al. assessed five psychological antecedents, each measured using three items: confidence (trust in vaccines and the systems that deliver them), complacency (perceived risk of vaccine-preventable diseases), constraints (perceived barriers to vaccination), calculation (preference for engaging in extensive information seeking and deliberation about vaccines), and collective responsibility (willingness to vaccinate to protect others) [18]. In this sample, the collective responsibility subscale did not demonstrate unidimensionality. Specifically, two of the three items failed to load on their intended factor and instead showed problematic cross-loadings onto factors representing distinct constructs, warranting exclusion of this subscale [19]. Internal consistency of the remaining four subscales was as follows: confidence α = 0.82, complacency α = 0.74, constraints α = 0.65, and calculation α = 0.80.

Vaccine conspiracy beliefs. The 7-item Vaccine Conspiracy Beliefs Scale (VCBS) by Shapiro et al. measured beliefs in vaccine-related conspiracy theories [20]. The current study adapted the VCBS, originally developed for parents, for use within a general adult population [21]. The internal consistency of the VCBS was α = 0.91 in the present study.

Descriptive vaccination norms. A 3-item scale adapted from prior research measured descriptive vaccination norms [22,23]. The scale assessed the extent to which participants agreed that most family and friends, community members, and Black adults in the United States receive vaccinations. The internal consistency of this scale was α = 0.73.

Injunctive vaccination norms. A 3-item scale adapted from prior research measured injunctive vaccination norms [22,23]. The scale assessed the extent to which participants agreed that most family and friends, community members, and Black adults in the United States would approve of their decision to vaccinate. Internal consistency was α = 0.85.

Trusted sources of vaccine information. A 9-item scale informed by prior research assessed how much participants trusted of various sources of vaccine information [24,25]. Responses were recorded on a 5-point scale ranging from 1 (not at all) to 5 (a lot). Psychometric testing identified three subscales: trust in traditional and social media (i.e., mass media, α = 0.82); healthcare providers and government health agencies (i.e., health institutions, α = 0.77); and family, close friends, and faith leaders (i.e., personal networks, α = 0.69).

Vaccine decision self-efficacy. The 11-item Vaccine Decision Self-Efficacy (VDSE) scale by Allen et al. assessed participants’ self-confidence in decision-making, including shared decision making [26]. Responses were recorded on a 5-point scale ranging from 1 (not at all confident) to 5 (very confident). The VDSE did not demonstrate unidimensionality in this sample and psychometric testing supported two subscales. The “obtain and understand” subscale (α = 0.90) comprised four items (e.g., “Get the facts about the risks of vaccines.”) and measured self-confidence to obtain and understand vaccine information for decision-making. The “participate” subscale (α = 0.92) included seven items and measured self-confidence to participate in shared decision-making (e.g., “Express your concerns about vaccines to a healthcare provider.”).

Sociodemographics. Measures included age, race and ethnicity, gender identity (0 = cisgender man, 1 = not cisgender man), education level (0 = some college or higher, 1 = high school or less), employment status (0 = employed, 1 = unemployed), estimated annual income (0 = ≥$25,000, 1 = <$25,000 or not sure), voting affiliation (0 = Democrat, 1 = Republican, Independent, or not registered to vote), religious affiliation (0 = Christian [e.g., Protestant, Catholic, Mormon, Jehovah’s Witness], 1 = other or none), healthcare insurance status, having a personal doctor [27], a measure of healthcare access (0 = yes, 1 = no), having seen a healthcare provider in the past year [28], a measure of healthcare utilization (0 = yes, 1 = no), having chronic medical conditions (0 = none, 1 = one or more), household composition (0 = lives with others, 1 = lives alone), current housing situation (0 = stable housing [i.e., living in one’s own home, permanent supportive housing, or school-affiliated dormitory], 1 = unstable housing), experienced homelessness in the past 6 months (0 = no, 1 = yes), internet access (0 = yes, 1 = no), and used government assistance in the past year (0 = no, 1 = yes).

Vaccination refusal. One item assessed participants’ past refusal of vaccines: “Have you ever refused getting certain vaccines for yourself?” Responses were coded as: 0 = no or not sure and 1 = yes.

COVID-19 vaccination. One item assessed vaccination against COVID-19: “Have you ever had a COVID-19 vaccine?” Responses were coded as: 0 = no or not sure and 1 = yes.

COVID-19 vaccination intention. One item assessed intention to receive an updated 2024–2025 COVID-19 vaccine: “Public health authorities at the Centers for Disease Control and Prevention (CDC) now recommend that everyone get an updated 2024–2025 COVID-19 vaccine. Do you plan to get an updated 2024–2025 COVID-19 vaccine?” The response options were: “Yes, I plan to get it as soon as possible”; “Yes, but I plan to wait before getting it”; “No”; and “I’m not sure.”

Annual influenza vaccination. One item assessed uptake of the annual 2023–2024 influenza vaccine: “During the past 12 months, have you had a flu shot?” Responses were coded as: 0 = no or not sure and 1 = yes.

Annual influenza vaccination intention. One item assessed intention to receive an annual 2024–2025 influenza vaccine: “Do you plan to get a flu shot this upcoming flu season?” The response options were: “Yes, I plan to get it as soon as possible”; “Yes, but I plan to wait before getting it”; “No”; and “I’m not sure.”

### 2.3. Statistical Analyses

Twelve indicators were included in the LPA: confidence; complacency; constraints; calculation; vaccine conspiracy beliefs; descriptive and injunctive vaccination norms; trust in media, health institutions, and personal networks for vaccine information; and decision self-efficacy to both obtain and understand vaccine information and participate in shared decision-making. Composite scores for the indicator variables were computed by averaging the item responses for each multi-item scale. Preliminary data screening involved checking that the indicators were normally distributed. The LPA and subsequent analyses were conducted using Mplus version 8.11.

The LPA followed four steps: model specification, identification, fit, and selection. To begin, a series of models with an increasing number of profiles (K) were specified and a maximum likelihood estimator with robust standard errors was used to estimate each model. Indicator variances were constrained to be equal across profiles, and covariances were fixed at zero within profiles [29]. Model identification was assessed using a three-stage optimization approach (i.e., 1000 sets of random starts in stage one, 500 best-performing sets in stage two, and 10 optimized sets in stage three). A model was deemed well-identified if the estimation process terminated normally and the best log-likelihood value replicated in at least 3% of the starting values [30].

Model fit was evaluated using the Akaike information criterion, Bayesian information criterion, and sample-size adjusted Bayesian information criterion, with lower values indicating better fit. Entropy was assessed as a classification index, with values equal to or greater than 0.80 indicating high profile separation [16]. The Bootstrapped Likelihood Ratio Test compared models with K and K-1 profiles, with a significant *p*-value indicating improved fit with the inclusion of an additional profile. Model selection balanced statistical fit with theoretical interpretability of the latent profiles [16]. For well-identified models, profile-specific parameter estimates (i.e., indicator means and standard errors and profile proportions) were examined, and only models in which all profiles comprised more than 5% of the total sample were considered as very small profile sizes may suggest overextraction [30].

Fifteen sociodemographic characteristics were evaluated as predictors of profile membership, with categorical variables dichotomized to avoid sparse cell sizes and facilitate robust model estimation [31]. Sociodemographic predictors of profile membership were examined using the R3STEP command. This command employs a three-step approach in which the latent profile measurement model and secondary multinominal logistic regression model are estimated independently to mitigate changes to the original profile structure [32]. In the current study, univariate models were estimated for each predictor variable, followed by an adjusted multivariate model that simultaneously included age, gender, and education a priori, along with any significant predictor identified in univariate analyses.

The DCAT command was used to evaluate the associations between latent profile membership and each of the five categorical vaccination outcomes. This command models the joint distribution of the latent profile variable and a categorical distal variable while preserving the original profile structure [32]. Mplus computes an overall test of association using Wald’s test, along with pairwise profile comparisons. Lastly, the DCAT command and related DCON command for continuous variables were used to describe the distribution of the 15 sociodemographic variables across latent profiles and identify characteristics significantly associated with profile membership.

## 3. Results

### 3.1. Sample Characteristics

Sociodemographic and vaccination characteristics are presented in Table 1. The final sample consisted of 203 Black adults with a mean age of approximately 34 years. Most participants identified as African American (75.9%), cisgender women (49.3%) and Democrats (45.8%). Overall, the sample reflected notable social and economic vulnerability: 41.4% reported a high school degree as their highest level of education attained, 34.5% reported an annual income less than $10,000, 45.8% experienced homelessness in the past 6 months, and 24.6% reported lacking internet access. Although more than half of study participants reported receiving a COVID-19 vaccine (61.1%), far less (28.6%) reported having vaccinated against influenza during the 2023–2024 season. The relatively high COVID-19 vaccination observed in this sample may be attributable to vaccine equity initiatives that occurred during the pandemic, including those implemented by the study partners, which may have increased access to COVID-19 vaccination in this neighborhood.

### 3.2. Identification of Vaccine Decision-Making Profiles

Table 2 summarizes the fit statistics for the latent profile models. Models with one to nine profiles were estimated. The nine-profile model failed to converge (i.e., under identified), and the five- to eight-profile models were not well-identified. A review of the information criteria suggested an evaluation of the two-, three-, and four-profile models. However, the four-profile model was excluded due to a very small profile size. The three-profile model showed improved fit compared to the two-profile model (*p* < 0.001). Interpreting the parameter estimates and considering theoretical interpretability, the three-profile model was selected and demonstrated a high degree of profile separation (i.e., entropy of 0.885).

Table 3 presents the parameter estimates for the three latent profiles. The largest profile (52.0%) was labeled Empowered and Engaged Trusters and consisted of individuals reporting high confidence (M = 3.97) and calculation (M = 4.03), alongside low complacency (M = 2.33) and constraints (M = 1.99). They also reported relatively low vaccine conspiracy beliefs (M = 2.52), relatively high descriptive (M = 3.62) and injunctive (M = 3.80) vaccination norms, and high trust in health institutions for vaccine information (M = 4.02), along with moderate trust in both mass media (M = 2.67) and personal networks (M = 3.10) for vaccine information. Their decision self-efficacy scores were high for both obtain and understand (M = 4.38) and participate (M = 4.45). Trusters recognize the threat posed by vaccine-preventable diseases and the utility of vaccination. They trust the institutions that develop, deliver, and regulate vaccines and view vaccines as safe and effective. Trusters view healthcare providers and government health agencies as credible sources of vaccine information. They also engage in extensive information-seeking and deliberate the risk of infection relative to the risk of vaccination to make a good decision. They are confident in their ability to make an informed vaccine decision on their own or with a healthcare provider and they do not generally perceive there to be barriers to vaccinate. Finally, Trusters see vaccination as common and socially approved by others in their social networks.

The second largest profile (32.2%), Unempowered and Unengaged Noncommitters, comprised individuals with mean scores near the midpoint across most indicators, including confidence (M = 3.18), complacency (M = 2.65), calculation (M = 3.10), conspiracy beliefs (M = 2.99), and descriptive (M = 2.89) and injunctive (M = 2.78) vaccination norms. These individuals reported relatively low vaccination constraints (M = 2.51), low trust in mass media (M = 2.24), and moderate trust in both health institutions (M = 2.66) and personal networks (M = 2.61). Overall, they reported moderate decision self-efficacy scores (M = 2.57 for obtain and understand and M = 2.75 for participate). Noncommitters lack strong convictions, expressing neutral views about the risk of infectious diseases and the safety of vaccines. They neither strongly believe nor disbelieve that those around them vaccinate or that others approve of them vaccinating. They also do not strongly endorse or outright reject conspiracy beliefs. Noncommitters do not trust the vaccine information presented in the media and instead place greater trust in information from personal networks and health institutions. While Noncommitters do not actively seek vaccine information or evaluate vaccination risks, they are not necessarily opposed to engaging in the decision-making process. While they believe barriers to vaccination are low, they do not feel very confident in their ability to make vaccine decisions.

The smallest profile (15.8%), labeled Empowered and Engaged Skeptics, was characterized by low confidence (M = 2.29) and constraints (M = 2.16), alongside high calculation (M = 4.29) and conspiracy beliefs (M = 3.91). These individuals also reported low trust in mass media (M = 1.58), health institutions (M = 2.09), and personal networks (M = 2.42). They reported relatively high scores for decision self-efficacy for obtain and understand (M = 3.57) and participate (M = 4.27). Their scores for complacency (M = 3.03) and descriptive (M = 3.40) and injunctive (M = 3.15) norms fell near the midpoint. Skeptics distrust vaccines and related institutions. They believe pharmaceutical companies and the government are fabricating and concealing information about vaccines from the public. Skeptics do not view vaccine information from healthcare providers, government health agencies, traditional media, social media, family, and friends as credible. Skeptics perceive low vaccination barriers and feel confident in their ability to both engage in shared decision-making with a healthcare provider and acquire and comprehend vaccine information on their own. They search for vaccine information and weigh the risks and benefits of vaccination. Lastly, Skeptics neither strongly believe nor disbelieve that vaccine-preventable diseases pose a threat, that vaccination is common, or that others approve of vaccination.

### 3.3. Profile Sociodemographic Characteristics

Table 4 summarizes the sociodemographic characteristics of the three latent profiles. Relative to Noncommitters and Skeptics, Trusters more often identified as cisgender men, Democrats, and Christians. Additionally, they reported more stable housing, less experiences with homelessness, and more internet access. Trusters reported more healthcare utilization (i.e., saw a healthcare provider in the past year) relative to the other profiles. Noncommitters more often reported as younger, less educated, and more unstably housed compared with Skeptics and Trusters. They more often reported having no chronic medical condition and no personal doctor, the latter indicating lower access to healthcare. Finally, a greater proportion of Skeptics identified as non-cisgender man (i.e., cisgender women or another gender identity), and had higher levels of education, employment, and income, suggesting higher socioeconomic status relative to the other profiles. Skeptics more often reported living alone, having at least one medical condition, and having a personal doctor. Results from the overall test showed that only age (χ^2^ = 7.771, *p* = 0.021), voting affiliation (χ^2^ = 11.042, *p* = 0.004), and healthcare utilization (χ^2^ = 6.624, *p* = 0.036) were significantly associated with profile membership.

### 3.4. Predictors of Profile Membership

Table 5 and Table 6 present findings from the univariate and multivariate multinomial logistic regression models, respectively. Results from the univariate analyses show that Noncommitters were significantly younger (OR = 0.946, 95% CI [0.904, 0.989]) and approximately three times more likely to be a Republican, Independent, or not registered to vote (OR = 3.176, 95% CI [1.578, 6.393]) and to not have seen a healthcare provider in the past year (OR = 2.729, 95% CI [1.316, 5.657]) relative to Trusters. Skeptics were about four times more likely to be a Republican, Independent, or not registered to vote relative to Trusters (OR = 3.954, 95% CI [1.463, 10.688]). Skeptics were less likely to have a low education level (i.e., high school degree or less; OR = 0.352, 95% CI [0.128, 0.967]) compared with Noncommitters.

Age, gender, education, voting affiliation, and healthcare utilization were simultaneously entered in the multivariate regression model. Younger age (aOR = 0.950, 95% CI [0.905, 0.996]) continued to predict being a Noncommitter relative to a Truster, as did being a Republican, Independent, or not registered to vote (aOR = 2.985, 95% CI [1.387, 6.427]) and not seeing a healthcare provider in the past year (aOR = 2.622, 95% CI [1.191, 5.771]). However, odds for the latter two predictors were slightly attenuated in the adjusted model. Skeptics reported higher odds of being a Republican, Independent, or not registered to vote (aOR = 5.393, 95% CI [1.843, 15.776]) relative to Trusters after controlling for age, gender, education, and healthcare utilization. Education level no longer predicted being a Skeptic relative to a Noncommitter in the adjusted model.

### 3.5. Vaccination Outcomes Across Profiles

Table 7 presents the distal outcomes across the three latent profiles. Overall, profile membership was significantly associated with past refusal of vaccination (*p* < 0.001), prior receipt of any COVID-19 vaccine (*p* < 0.001), intention to receive the updated 2024–2025 COVID-19 vaccine (*p* < 0.001), prior receipt of the 2023–2024 annual influenza vaccine (*p* = 0.001), and intention to receive the 2024–2025 annual influenza vaccine (*p* < 0.001). Skeptics reported the highest past vaccination refusal (92.2%), followed by Trusters (35.7%) and Noncommitters (34.7%), with significant differences identified between Skeptics and both Trusters (*p* < 0.001) and Noncommitters (*p* < 0.001). Trusters reported the highest past uptake of COVID-19 vaccines (77.9%), followed by Noncommitters (50.3%) and Skeptics (29.1%), with significant differences between Trusters and both Noncommitters (*p* = 0.001) and Skeptics (*p* < 0.001). A similar pattern was observed for the 2023–2024 influenza vaccine, with uptake differing among Trusters (38.4%), Noncommitters (23.3%), and Skeptics (7.5%). However, the only significant difference was detected between Trusters and Skeptics for this outcome (*p* < 0.001). Lastly, intention to receive the updated COVID-19 and 2024–2025 influenza vaccines was highest among Trusters (32.7% and 39.9%, respectively), lowest among Skeptics (0.00% and 0.00%, respectively), with Noncommitters falling between them (17.3% and 6.1%, respectively).

## 4. Discussion

Black populations are uniformly portrayed as vaccine-hesitant, a label that obscures heterogeneity in vaccine decision-making. In the present study, an LPA was employed to identify distinct and meaningful profiles of vaccine decision-making among Black adults in South Central Los Angeles. Guided by the socioecological and 5C frameworks, the profiles differed across 12 indicators: confidence; complacency; constraints; calculation; vaccine conspiracy beliefs; descriptive and injunctive vaccination norms; trust in health institutions, mass media, and personal networks for vaccine information; and decision self-efficacy to both obtain and understand vaccine information and participate in shared decision-making. Three latent profiles emerged: Empowered and Engaged Trusters (52.0%), Unempowered and Unengaged Noncommitters (32.2%), and Empowered and Engaged Skeptics (15.8%). Secondary analyses identified healthcare utilization, age, and voting affiliation as significant predictors of profile membership and also showed that general, COVID-19, and influenza vaccination outcomes aligned with the psychological profile configurations in expected ways. Study findings add to limited research on vaccine decision-making among Black adults, offering insight into not only within-group diversity in vaccine acceptance, but also why acceptance may vary in this population. Findings have implications for tailored interventions to promote vaccine acceptance and uptake.

Individuals in the Empowered and Engaged Trusters profile reported high confidence, high trust in health institutions, low conspiracy beliefs, complacency, and constraints, high calculation and decision self-efficacy, relatively high vaccination norms, and moderate trust in media and personal networks. This profile reflects a configuration characterized by positive vaccine attitudes and beliefs, an information environment anchored in official sources, active engagement in the decision-making process, a supportive social normative context, and a perceived capacity to make vaccination decisions. These characteristics suggest that Trusters are generally vaccine-accepting. One plausible pathway underlying this profile is that institutional trust directs Trusters’ information seeking toward healthcare providers and government health agencies who are perceived as credible [33]. Utilizing these official sources may increase exposure to evidence-based vaccine information and support a deliberation process that fosters confidence [18]. Trust in health institutions may also reduce susceptibility to misinformation and conspiracy beliefs, promoting positive attitudes [34]. High self-efficacy may further motivate information seeking and enable Trusters to engage in shared decision-making with healthcare providers, strengthening confidence via decisional support [35]. Moreover, low perceived constraints to vaccination may facilitate translating decisions into behavior. Finally, strong normative beliefs may serve to reinforce vaccine confidence through social validation [36]. Overall, this profile underscores the role of institutional trust in decision-making and mutually reinforcing relationships that may foster acceptance.

The Unempowered and Unengaged Noncommitters profile was characterized by midpoint scores across confidence, complacency, calculation, conspiracy beliefs, descriptive and injunctive vaccination norms alongside relatively low constraints, low trust in mass media and moderate trust in health institutions and personal networks for vaccine information, and moderate decision self-efficacy. These characteristics reflect not only vaccine apathy, characterized by weak attitudes and limited engagement in the decision-making process [37], but apathy that coincides with limited self-confidence to make vaccine decisions. For Noncommitters, limited self-efficacy to obtain and understand information may reduce motivation to engage in information seeking and deliberation. Self-efficacy shapes motivation, effort, and persistence in information seeking such that individuals with limited self-efficacy may be less likely to engage in cognitively demanding tasks like deliberation of vaccination risks [38]. Weak vaccine attitudes, which can contribute to low engagement in decision-making [39], may be reinforced in the absence of strong normative beliefs. Despite moderate trust in health institutions for information, their perceived credibility may be insufficient to motivate information seeking if self-confidence is lacking.

Those in the Empowered and Engaged Skeptics profile reported low confidence and constraints, high calculation and conspiracy beliefs, low trust in mass media, health institutions, and personal networks for vaccine information, high decisional self-efficacy overall, with complacency and descriptive and injunctive norms near the midpoint. This profile was characterized by distrust, negative vaccine attitudes and beliefs, weak normative beliefs, and high decisional engagement and perceived capacity for decision-making. This configuration suggests vaccine-refusal rooted in skepticism. Within the Black community, vaccine skepticism is often linked to a justifiable distrust rooted in individual and collective experiences of racism, exploitation, and marginalization [40]. Skeptics’ low trust in various sources of information also suggests a more generalized form of distrust. In the presence of this distrust, Skeptics may actively seek information from alternative and unofficial sources that align with their pre-existing beliefs, amplifying skepticism through confirmation bias [41]. Within this context, Skeptics’ deliberation process may reinforce low vaccine confidence and institutional distrust. Additionally, conspiracy beliefs may function as an interpretive lens that informs source credibility and the interpretation of risks, negatively shaping vaccine attitudes [21]. Despite confidence to participate in shared decision-making with a healthcare provider, distrust may impede such interactions. Finally, skepticism may be fostered in the absence of strong social counterpressure.

Healthcare utilization, age, and voting affiliation predicted profile membership in the adjusted model. Compared with Trusters, Noncommitters were less likely to have seen a healthcare provider in the past year, indicating lower healthcare utilization. Healthcare utilization represents a point of contact with healthcare providers, who are important institutional-level influencers of vaccine decisions, with provider recommendations reported as strong predictors of vaccine acceptance and uptake [42,43]. Lower healthcare use among Noncommitters suggests fewer opportunities to receive vaccination services, provider recommendations, vaccine information, and support via shared decision-making [44]. Alternatively, limited self-confidence to engage in shared decision-making may lessen engagement with the healthcare system more broadly [45]. While age and health status are key determinants of healthcare use, medical mistrust, access barriers, and challenges navigating the healthcare system may also play a role, particularly in lower-income and Black communities [46,47]. In the present study, age was controlled for in the adjusted model while healthcare access and chronic health status were not significantly associated with profile membership in univariate analyses. Given inequities in medically underserved communities, healthcare access may influence utilization beyond the individual-level access measure employed in this study.

Noncommitters were more likely to be younger relative to Trusters. Although the age difference was modest, younger age was associated with a profile characterized by weak cognitions, limited engagement in the decision-making process, and modest decision self-efficacy, even after adjustment for healthcare use. This finding suggest that younger participants were more likely to be apathetic toward vaccines and feel less capable of making informed decisions. Prior studies have reported mixed associations between age and vaccine acceptance in Black populations [11,48], while research on age and vaccine apathy is lacking. Notably, Mancuso et al. found that younger age was associated with a vaccine-receptive rather than a vaccine-neutral profile among Black adults [13]. However, their study identified attitudinal profiles, limiting direct comparison. One possible explanation is that, because self-efficacy may develop through mastery and vicarious experiences, younger adults may have fewer opportunities to build confidence [49]. This observation has implications for promoting vaccine acceptance across the life span, with attention to addressing apathy and strengthening decision self-efficacy among younger Black adults. Further research is needed to investigate this association and the underlying mechanisms.

Compared with Trusters, both Skeptics and Noncommitters were more likely to identify as a Republican, Independent, or not registered to vote. Because these categories were combined to improve model estimation in this smaller sample, it is unclear whether the observed associations are due to a particular political party affiliation or voter registration status. Consequently, the relationship between these categories and vaccine decision-making cannot be determined. Prior research has identified non-Democratic party affiliation as a robust predictor of low vaccine acceptance, with individuals reporting lower institutional and vaccine confidence, greater exposure to vaccine-critical information, and increased susceptibility to conspiracy beliefs [50,51]. In contrast, vaccine-related research examining voter registration status is lacking. Among Black Americans, Republican and Independent party affiliations and lack of voter registration have been linked to disillusionment with the majority Democratic Party and voting disenfranchisement, respectively [52]. Additional research is needed to determine whether political party affiliation or voter registration status is associated with vaccine decision-making processes reflecting both engaged skepticism and unempowered apathy among Black adults in disinvested communities.

Profile membership was significantly associated with all five vaccination outcomes, including past refusal of vaccination, past COVID-19 and annual influenza vaccination, and future intentions to receive updated COVID-19 and annual influenza vaccines. Overall, the vaccination outcomes varied across the three profiles of decision-making in expected directions with Trusters reporting greater vaccination intentions and uptake than both Skeptics and Noncommitters. Skeptics generally reported lower vaccination outcomes than Noncommitters. Together, these findings provide validation to the latent profile measurement model by showing alignment between vaccine decision-making, vaccination intentions, and vaccination behaviors.

### 4.1. Theoretical Contributions

This study adds to a limited body of vaccine research conducting LPA in Black adults [13,14], and is the first to use an integrated socioecological and 5C framework. By employing a person-centered approach, profile configurations reveal how constellations of vaccine attitudes and beliefs, trust in information sources, vaccination norms, engagement in the decision-making process, and decision self-efficacy co-occur within Black adults to shape vaccine decision-making and acceptance. While the 5C collective responsibility subscale was excluded from the LPA due to suboptimal psychometric performance in this sample, its exclusion should not be interpreted as evidence that this construct does not shape decision-making. In fact, Thier et al. found collective responsibility to be associated with positive vaccine attitudes among Black Americans [53]. Instead, the performance of this subscale may vary by setting, with Thier et al. also reporting issues and calling for its examination across populations and vaccine type. In past LPA studies conducted primarily among healthcare workers and in international settings, collective responsibility distinguished profiles of vaccine acceptance [54,55]. However, given these population differences, it’s unclear whether collective responsibility functions similarly among Black adults in the U.S., though its role suggests it may be a relevant dimension of vaccine decision-making requiring further study in this population.

Findings reveal meaningful heterogeneity in vaccine decision-making among Black adults in a historically disinvested and medically underserved community, suggesting distinct pathways underlying vaccine acceptance. Institutional trust distinguished Trusters from Skeptics, underscoring its importance in shaping vaccine decision-making [40]. Both Trusters and Skeptics reported high calculation and decision self-efficacy despite opposing configurations of attitudes, beliefs, and trust in information sources. This aligns with research suggesting that calculation (i.e., extensive information seeking and deliberation of vaccination risks) depends on trust and the information source, potentially leading to differences in acceptance [18,56]. Additionally, high decision self-efficacy among Skeptics suggests that self-confidence to obtain and understand information and participate in shared decision-making does not necessarily translate to vaccine acceptance or uptake, particularly when low perceived credibility of health institutions, low vaccine confidence, and belief in conspiracy theories are present. Noncommitters were characterized by the co-occurrence of moderate institutional trust, weak attitudes and beliefs, limited engagement, and moderate self-confidence, revealing a relationship between decision self-efficacy and vaccine apathy not previously reported in the literature. Low perceived constraints across all three profiles should be interpreted cautiously as this does not suggest that structural access barriers are absent, but rather that perceived barriers to vaccination did not differentiate decision-making in this sample. This may be due to perceived access to vaccination outside of traditional clinical settings and may reflect selection bias resulting from the study’s community-based recruitment strategy.

By uncovering heterogeneity in decision-making, this study provides insight into within-group diversity in vaccine acceptance and advances current understanding of vaccine apathy. The predominance of Trusters in the sample is consistent with data suggesting that most U.S. adults are accepting of vaccines, challenging portrayals of Black populations as uniformly vaccine-hesitant [57]. Relatedly, the high proportion of Noncommitters in the sample draws attention to vaccine apathy as a potentially important yet understudied dimension of vaccine acceptance among Black adults. Whether this apathy reflects genuine indifference, disinterest, or unawareness is unclear and requires additional research. Currently, vaccine apathy may be underestimated in the literature due to social desirability bias, lack of validated measures, and conflation with hesitancy [37]. Unlike vaccine hesitancy, which has traditionally been conceptualized along an attitudinal or behavioral continuum between acceptance and refusal, vaccine apathy encompasses both attitudinal and engagement dimensions [37], and should not be interpreted as the midpoint on such continua. This study supports conceptualizations of vaccine acceptance that move beyond a unidimensional continuum and incorporate the decision-making process [58]. Distinguishing vaccine apathy is relevant for addressing lower vaccination coverage in Black communities and suggests that efforts may benefit from fostering decisional self-efficacy and engagement alongside strengthening institutional trust and vaccine confidence.

Analyses of sociodemographic predictors and vaccination outcomes further contextualize decision-making and support validity of the measurement model, respectively. The study highlights the importance of considering healthcare utilization alongside access in underserved Black communities, as use facilitates contact with providers who strongly influence vaccine decision-making. This finding advances understanding of vaccine apathy by identifying healthcare utilization as an institutional-level factor that may both shape and reflect weak vaccine attitudes, limited decisional engagement, and lower self-efficacy among Noncommitters. Beyond healthcare factors, observed age-related differences may reflect how individuals approach decision-making, underscoring lower self-efficacy as a potentially important co-occurring feature of vaccine apathy among younger Black adults. Voting affiliation, which distinguished engaged vaccine skepticism from unempowered vaccine apathy, may highlight political heterogeneity present in Black communities, although the respective roles of political affiliation and voter registration remain unclear and warrants further study. Notably, sociodemographic predictors reflecting the broader context of a historically disinvested community, including housing instability, lower income, and limited internet access, were not associated with profile membership, possibly because social and economic disadvantage was reported uniformly across the sample. This observation does not imply that structural factors are unrelated to vaccine decision-making, but that distinct psychological patterns emerged among Black adults within a shared context of disinvestment. Lastly, the study contributes to limited research employing LPA, demonstrating that it can be used to capture the multidimensional nature of vaccine acceptance and identify decision-making profiles meaningfully associated with vaccination intentions and behaviors.

### 4.2. Implications for Public Health Interventions

Study findings inform interventions to promote vaccine acceptance and uptake within historically disinvested Black communities. The profile-specific approaches discussed below emphasize tailored communication and engagement strategies for each subgroup. Consistent with the socioecological framework, these interventions should be implemented as part of a multilevel approach alongside policies to expand access to vaccination services in both clinical and non-traditional settings, including strengthening local healthcare infrastructure and utilizing trusted community-based partnerships [5]. Health institutions and medical schools must address institutional racism and build trust by increasing the representation of Black healthcare providers, implementing anti-racism training, and ensuring providers are equipped to deliver strong vaccine recommendations, engage in shared clinical decision-making, and address misinformation in ways that are culturally responsive to Black adults [59,60]. Importantly, reforms to dismantle systemic racism, abolish discriminatory practices, and eliminate existing inequities must be prioritized to repair trust more broadly and improve the social conditions in which vaccine decision-making occurs.

For Empowered and Engaged Trusters, interventions should prioritize maintaining acceptance across vaccines by leveraging institutional trust, reinforcing confidence, and supporting informed decision-making. Efforts should employ healthcare providers as trusted messengers to provide strong vaccine recommendations, timely evidence-based information, and shared decision-making that reinforces trust in vaccines and the systems that delivers them [44]. Messaging from healthcare professionals and public health agencies should emphasize vaccine safety and effectiveness, the risks of vaccine-preventable diseases, and the supportive vaccination norms already evident within this subgroup.

Interventions for Unempowered and Unengaged Noncommitters should focus on addressing vaccine apathy, strengthening self-efficacy, and fostering trusted connections to healthcare sources to prevent drifts toward non-acceptance. Outreach efforts should not rely solely on clinical encounters or passive public health messaging. Instead, healthcare providers, vaccine information, and vaccination services should be brought into trusted community settings via mobile clinics and other community-based delivery models to increase opportunities for provider recommendations, shared decision-making, and vaccination. Trusted messengers should proactively engage younger Black adults. Decision aids and vaccine literacy initiatives can strengthen confidence to make informed vaccine decisions [61,62]. Interventions that promote patient empowerment may also help build institutional trust [59]. Communication strategies for vaccine apathy, should include messages that are catchy, simple, and rely on emotion, humor, or heuristics to reduce cognitive load and information processing [37]. Messaging should utilize engaging formats such as visuals, short videos, and infographics, and be delivered in everyday places by familiar sources that reflect salient social in-groups [37].

Lastly, for Empowered and Engaged Skeptics, interventions should center on repairing institutional trust, directing information seeking toward credible sources, and reducing skepticism to overcome vaccine refusal. Alongside institutional reforms to earn and repair trust within Black communities, healthcare and public health institutions must invest in sustained partnerships with trusted community leaders, organizations, and racially concordant providers to bring vaccine information and decisional support to community settings [5,63]. Efforts are also needed to identify specific trusted messengers and communication channels among Skeptics. Communication strategies should leverage Skeptics’ high self-confidence and engagement through transparent, nonjudgmental dialogue that respects autonomy, acknowledges the historical and ongoing effects of racism and medical injustice, and addresses vaccination concerns [5,40]. Preemptive inoculation strategies, which proactively expose misinformation tactics, have been shown to prevent belief in new conspiracy theories [64]. Additionally, active inoculation strategies that teach critical evaluation skills can promote long-term resilience against misinformation [64]. Lastly, increasing vaccine literacy skills may weaken endorsement of conspiracy theories among Black adults [65].

### 4.3. Study Limitations

Several limitations should be considered when interpreting study findings. First, the use of nonprobability sampling limits generalizability to other populations. Second, the cross-sectional design precludes causal inference and the temporal ordering of study variables. Third, the modest sample size (N = 203) may have constrained statistical power to detect less prevalent subgroups and associations in the regression models. Notably, there is no universal minimum sample size for LPA, and power depends not only on sample size but indicator quality and profile separation [66]. To mitigate this limitation, the study utilized psychometrically evaluated indicators and applied rigorous model fit procedures, supporting selection of a well-identified, parsimonious model. Fourth, all study measures were self-reported and thus subject to recall and social desirability bias. The study may also be subject to selection bias as previously noted. Fifth, several measurement limitations warrant consideration. Namely, the 5C collective responsibility subscale was excluded from the LPA precluding its evaluation as a determinant of vaccine decision-making, and the voting affiliation variable was dichotomized, obscuring distinctions between political party affiliation and voter registration status. Finally, study findings may not translate to decision-making about specific vaccines.

### 4.4. Future Research Directions

Reproducibility studies are needed to determine whether similar latent profiles emerge among Black adults in other geographic settings or within other marginalized populations. Future studies should utilize larger, probability-based samples to improve statistical power and generalizability of the findings. Longitudinal studies can be employed to examine the stability of the latent profiles and monitor changes in vaccine decision-making over time. Importantly, research is needed to investigate the association between voting affiliation and vaccine decision-making as well as the role of collective responsibility in decision-making. Qualitative research approaches, such as in-depth interviews, may provide rich insights into the relationships observed in this study. Finally, studies should incorporate other decisional constructs, such as decisional conflict as well as validated measures of vaccine apathy to deepen understanding of vaccine decision-making and acceptance among Black adult populations.

## 5. Conclusions

The present study underscores the heterogeneity of vaccine decision-making among Black adults in a historically disinvested and medically underserved community by identifying distinct patterns of vaccine-related attitudes and beliefs, trust in information sources, decisional engagement, and decision self-efficacy. These patterns clarify how vaccine acceptance may emerge through different pathways within a shared structural context, challenging broad characterizations of Black adults as uniformly vaccine-hesitant, and distinguishing vaccine acceptance, apathy, and refusal. Findings can inform tailored strategies that maintain confidence among Trusters, strengthen decisional engagement and self-efficacy among Noncommitters, and address institutional distrust and skepticism among Skeptics. Implemented alongside efforts to expand vaccination access, repair institutional trust, and address structural inequities, these approaches may more effectively promote vaccine acceptance and uptake in historically disinvested Black communities.

## Figures and Tables

**Table 1:** Sociodemographic and vaccination characteristics of the sample (N = 203).

Variable	n (%)
Age in years, M (SD)	33.85 (7.54)
Race and ethnicity	
Black African American	154 (75.86)
Black African	8 (3.94)
Black Caribbean	4 (1.97)
Black multiracial or multiethnic	37 (18.23)
Gender identity	
Cisgender man	98 (48.28)
Cisgender woman	100 (49.26)
Transgender man	1 (0.49)
Transgender woman	1 (0.49)
Other	3 (1.48)
Education level	
Less than high school	19 (9.36)
High school or GED	84 (41.38)
Some college, associate degree, or trade school	63 (31.03)
Bachelor’s degree or higher	37 (18.23)
Employment status	
Employed full-time	75 (36.95)
Employed part-time	35 (17.24)
Unemployed, homemaker, or stay-at-home caregiver	2 (0.99)
Unemployed, retired	2 (0.99)
Unemployed, student	12 (5.91)
Unemployed, unable to work	16 (7.88)
Unemployed, other	61 (30.05)
Estimated annual income	
$0–$9999	70 (34.48)
$10,000–$14,499	23 (11.33)
$15,000–$24,999	10 (4.93)
$25,000–$34,999	19 (9.36)
$35,000–$49,999	17 (8.37)
$50,000–$74,999	14 (6.90)
$75,000 or more	15 (7.39)
Not sure	35 (17.24)
Voting affiliation	
Democrat	93 (45.81)
Republican	18 (8.87)
Independent	54 (26.60)
Not registered to vote	38 (18.72)
Religious affiliation	
Christian	136 (67.00)
Muslim	8 (3.94)
Hindu	1 (0.49)
Jewish	1 (0.49)
Buddhist	0 (0.00)
Agnostic	15 (7.39)
Atheist	5 (2.46)
Other	37 (18.23)
Healthcare insurance	
Public (e.g., Medicaid or Medicare)	130 (64.04)
Private (e.g., HMO or PPO)	49 (24.14)
Veterans Affairs or military	5 (2.46)
No healthcare insurance	19 (9.36)
Healthcare access	
Yes	118 (58.13)
No	85 (41.87)
Healthcare utilization (past year)	
Yes	145 (71.43)
No	58 (28.57)
Chronic medical conditions ^a^	
Mental health condition	44 (21.67)
High blood pressure	38 (18.72)
Obesity or overweight	18 (8.87)
Chronic lung disease	13 (6.40)
Diabetes (type 1 or 2)	11 (5.42)
Substance abuse	7 (3.45)
Chronic heart condition	5 (2.46)
Cancer	3 (1.48)
HIV or AIDS	3 (1.48)
Chronic liver disease	1 (0.49)
Sickle cell disease	1 (0.49)
Tuberculosis	1 (0.49)
Chronic kidney disease	1 (0.49)
Other medical condition	20 (9.85)
No medical condition	100 (49.26)
Household composition	
Lives alone	74 (36.45)
Lives with others	129 (63.55)
Current housing situation	
Own home or apartment	108 (53.20)
Permanent supportive housing	3 (1.48)
School or university dorm	2 (0.99)
Temporarily staying with others	31 (15.27)
Shelter	29 (14.29)
Hotel or motel	11 (5.42)
Transitional housing, sober living, or safe haven	8 (3.94)
Outside or abandoned building	8 (3.94)
Car, truck, or RV	3 (1.48)
Experienced homelessness (past 6 months)	
Yes	93 (45.81)
No	110 (54.19)
Internet access	
Yes	153 (75.37)
No	50 (24.63)
Government assistance (past year)	
Yes	88 (43.35)
No	115 (56.65)
Vaccination refusal	
Yes	93 (45.81)
No	96 (47.29)
Not sure	14 (6.90)
COVID-19 vaccination	
Yes	124 (61.08)
No	76 (37.44)
Not sure	3 (1.48)
Annual influenza vaccination	
Yes	58 (28.57)
No	136 (67.00)
Not sure	9 (4.43)
COVID-19 vaccination intention	
Yes, I plan to get it as soon as possible	46 (22.66)
Yes, but I plan to wait before getting it	28 (13.79)
No	80 (39.41)
Not sure	49 (24.14)
Annual influenza vaccination intention	
Yes, I plan to get it as soon as possible	45 (22.17)
Yes, but I plan to wait before getting it	41 (20.20)
No	83 (40.89)
Not sure	34 (16.75)

Table notes. n = number; M = mean; SD = standard deviation. ^a^ Percentages do not sum to 100% as participants were asked to select all that apply.

**Table 2:** Fit statistics for the estimated latent profile models.

Profiles	AIC	BIC	SA-BIC	Entropy	BLRT *p*	Smallest Profile (%)
1	6934.180	7013.697	6937.659	-	-	-
2	6603.981	6726.570	6609.344	0.847	<0.001 ^c^	45.61
**3**	**6493.871**	**6659.532**	**6501.119**	**0** **.885**	**<0.001**	**15.77**
4	6398.403	6607.135	6407.535	0.906	<0.001	3.97
5 ^a^	6324.055	6575.859	6335.072	0.876	<0.001	3.96
6 ^a^	6266.420	6561.295	6279.321	0.891	<0.001 ^c^	4.03
7 ^a^	6212.583	6550.530	6227.369	0.904	<0.001	1.97
8 ^a^	6171.568	6552.587	6188.238	0.916	<0.001 ^c^	1.96
9 ^b^	6134.695	6558.785	6153.249	0.927	<0.001 ^c^	0.99

Table notes. AIC = Akaike information criterion; BIC = Bayesian information criterion; SA-BIC = sample size-adjusted Bayesian information criterion; BLRT = Bootstrap Likelihood Ratio Test. ^a^ The model was not well-identified. ^b^ The model did not converge. ^c^ The *p*-value was reported as potentially untrustworthy due to local maxima. Bold values reflect the selected model.

**Table 3:** Parameter estimates for the selected three-profile model.

**Variable**	**Trusters**		**Noncommitters**		**Skeptics**	
	52.0%		32.2%		15.8%	
	N = 106		N = 65		N = 32	
	**M**	**SE**	**M**	**SE**	**M**	**SE**
Confidence	3.97	0.07	3.18	0.13	2.29	0.21
Complacency	2.33	0.10	2.65	0.11	3.03	0.24
Constraints	1.99	0.09	2.51	0.12	2.16	0.24
Calculation	4.03	0.08	3.10	0.19	4.29	0.12
Vaccine conspiracy beliefs	2.52	0.10	2.99	0.14	3.91	0.18
Descriptive vaccination norms	3.62	0.09	2.89	0.15	3.40	0.21
Injunctive vaccination norms	3.80	0.10	2.78	0.15	3.15	0.19
Trust in mass media for vaccine information	2.67	0.12	2.24	0.16	1.58	0.12
Trust in health institutions for vaccine information	4.02	0.09	2.66	0.16	2.09	0.23
Trust in personal networks for vaccine information	3.10	0.12	2.61	0.15	2.42	0.25
Vaccine decision self-efficacy (obtain and understand)	4.38	0.09	2.57	0.11	3.57	0.32
Vaccine decision self-efficacy (participate)	4.45	0.08	2.75	0.15	4.27	0.23

Table notes. M = mean; SE = standard error.

**Table 4:** Sociodemographic characteristics of the three latent profiles.

**Variable**	**Trusters**	**Noncommitters**	**Skeptics**	**Overall Test**	
	N = 106	N = 65	N = 32	χ^2^	*p*
Age (years)	34.78 (0.70)	31.65 (0.98)	35.01 (1.23)	**7.771**	**0** **.021**
Gender identity				2.517	0.284
Cisgender man	0.527 (0.05)	0.474 (0.07)	0.349 (0.10)		
Not cisgender man	0.473 (0.05)	0.526 (0.07)	0.651 (0.10)		
Education level				4.064	0.131
Some college or higher	0.523 (0.05)	0.378 (0.07)	0.621 (0.11)		
High school or less	0.477 (0.05)	0.622 (0.07)	0.379 (0.11)		
Employment status				1.063	0.588
Employed	0.567 (0.05)	0.464 (0.09)	0.613 (0.10)		
Unemployed	0.433 (0.05)	0.536 (0.09)	0.387 (0.10)		
Estimated annual income				2.843	0.241
≥$25,000	0.359 (0.05)	0.237 (0.06)	0.362 (0.10)		
<$25,000 or not sure	0.641 (0.05)	0.763 (0.06)	0.638 (0.10)		
Voting affiliation				**11.042**	**0** **.004**
Democrat	0.619 (0.08)	0.344 (0.07)	0.211 (0.11)		
Republican, Independent, or not registered to vote	0.381 (0.08)	0.656 (0.07)	0.789 (0.11)		
Religious affiliation				1.435	0.488
Christian	0.712 (0.05)	0.622 (0.07)	0.627 (0.11)		
Other or none	0.288 (0.05)	0.378 (0.07)	0.373 (0.11)		
Healthcare access				1.857	0.395
Yes	0.600 (0.05)	0.512 (0.07)	0.663 (0.10)		
No	0.400 (0.05)	0.488 (0.07)	0.337 (0.10)		
Healthcare utilization (past year)				**6.624**	**0** **.036**
Yes	0.785 (0.04)	0.582 (0.07)	0.752 (0.09)		
No	0.215 (0.04)	0.418 (0.07)	0.248 (0.09)		
Chronic medical conditions				0.071	0.965
None	0.486 (0.05)	0.509 (0.07)	0.480 (0.12)		
One or more	0.514 (0.05)	0.491 (0.07)	0.520 (0.12)		
Household composition				1.326	0.515
Lives with others	0.674 (0.05)	0.622 (0.07)	0.535 (0.13)		
Lives alone	0.326 (0.05)	0.378 (0.07)	0.465 (0.13)		
Current housing situation				3.158	0.206
Stable housing	0.614 (0.05)	0.456 (0.07)	0.571 (0.10)		
Unstable housing	0.386 (0.05)	0.544 (0.07)	0.429 (0.10)		
Experienced homelessness (past 6 months)				0.36	0.835
No	0.565 (0.05)	0.514 (0.07)	0.523 (0.10)		
Yes	0.435 (0.05)	0.486 (0.07)	0.477 (0.10)		
Internet access				0.493	0.781
Yes	0.777 (0.04)	0.727 (0.07)	0.731 (0.10)		
No	0.223 (0.04)	0.273 (0.07)	0.269 (0.10)		
Government assistance (past year)				2.191	0.334
No	0.562 (0.05)	0.642 (0.07)	0.438 (0.10)		
Yes	0.438 (0.05)	0.358 (0.07)	0.562 (0.10)		

Table notes. For age, a continuous variable, the DCON command returned the mean and standard error. For the remaining categorical variables, the DCAT command returned probabilities and standard errors. Standard errors are reported in parentheses. The overall test had 2 degrees of freedom. Bold values indicate statistically significant results with an alpha level of 0.05.

**Table 5:** Results for univariable logistic regression.

Variable	Noncommitters vs. Trusters (ref)			Skeptics vs. Trusters (ref)			Skeptics vs. Noncommitters (ref)		
	OR	SE	95% CI	OR	SE	95% CI	OR	SE	95% CI
Age (years)	**0.946**	**0.022**	**0.904, 0.989**	0.995	0.031	0.936, 1.059	1.053	0.036	0.984, 1.126
Gender identity									
Cisgender man (ref)									
Not cisgender man	1.292	0.437	0.666, 2.505	1.846	0.865	0.737, 4.623	1.429	0.724	0.529, 3.858
Education level									
Some college or higher (ref)									
High school or less	1.832	0.634	0.930, 3.609	0.646	0.304	0.257, 1.626	**0.352**	**0.182**	**0.128, 0.967**
Employment status									
Employed (ref)									
Unemployed	1.303	0.440	0.672, 2.526	0.747	0.350	0.298, 1.870	0.573	0.290	0.213, 1.546
Estimated annual income									
≥$25,000 (ref)									
<$25,000 or not sure	1.741	0.659	0.830, 3.655	1.190	0.573	0.463, 3.059	0.683	0.371	0.236, 1.981
Voting affiliation									
Democrat (ref)									
Republican, Independent, or not registered to vote	**3.176**	**1.134**	**1.578, 6.393**	**3.954**	**2.006**	**1.463, 10.688**	1.245	0.689	0.421, 3.684
Religious affiliation									
Christian (ref)									
Other or none	1.294	0.462	0.643, 2.605	1.276	0.611	0.499, 3.261	0.986	0.507	0.360, 2.700
Healthcare access									
Yes (ref)									
No	1.572	0.535	0.807, 3.063	0.825	0.394	0.323, 2.106	0.525	0.269	0.192, 1.435
Healthcare utilization (past year)									
Yes (ref)									
No	**2.729**	**1.015**	**1.316, 5.657**	1.558	0.808	0.564, 4.303	0.571	0.304	0.201, 1.620
Chronic medical conditions									
None (ref)									
One or more	0.910	0.307	0.470, 1.762	1.012	0.461	0.415, 2.470	1.113	0.550	0.423, 2.930
Household composition									
Lives with others (ref)									
Live alone	1.198	0.423	0.600, 2.392	1.735	0.802	0.702, 4.292	1.448	0.724	0.544, 3.858
Current housing situation									
Stable housing (ref)									
Unstable housing	1.902	0.650	0.974, 3.717	1.113	0.516	0.449, 2.761	0.585	0.293	0.219, 1.560
Experienced homelessness (past 6 months)									
No (ref)									
Yes	1.185	0.401	0.611, 2.299	1.221	0.556	0.500, 2.983	1.030	0.509	0.391, 2.713
Internet access									
Yes (ref)									
No	1.259	0.488	0.589, 2.691	1.185	0.621	0.424, 3.312	0.942	0.528	0.314, 2.824
Government assistance (past year)									
No (ref)									
Yes	0.794	0.274	0.404, 1.562	1.435	0.655	0.586, 3.511	1.806	0.903	0.678, 4.810

Table notes. OR = odds ratio; SE = standard error; CI = confidence interval; ref = reference group. Statistical significance was based on a 95% CI that did not span 1.00. Bold values indicate significant results.

**Table 6:** Results for multivariable logistic regression.

Variable	Noncommitters vs. Trusters (ref)			Skeptics vs. Trusters (ref)			Skeptics vs. Noncommitters (ref)		
	aOR	SE	95% CI	aOR	SE	95% CI	aOR	SE	95% CI
Age (years)	**0.950**	**0.023**	**0.905, 0.996**	0.995	0.036	0.928, 1.067	1.048	0.039	0.974, 1.128
Gender identity									
Cisgender man (ref)									
Not cisgender man	1.639	0.613	0.787, 3.413	2.355	1.213	0.858, 6.462	1.437	0.792	0.488, 4.230
Education level									
Some college or higher (ref)									
High school or less	1.096	0.425	0.513, 2.343	0.380	0.219	0.122, 1.178	0.346	0.207	0.108, 1.114
Voting affiliation									
Democrat (ref)									
Republican, Independent, or not registered to vote	**2.985**	**1.168**	**1.387, 6.427**	**5.393**	**2.954**	**1.843, 15.776**	1.806	1.075	0.563, 5.800
Healthcare utilization (past year)									
Yes (ref)									
No	**2.622**	**1.055**	**1.191, 5.771**	1.958	1.235	0.569, 6.740	0.747	0.485	0.209, 2.668

Table notes. aOR = adjusted odds ratio; SE = standard error; CI = confidence interval; ref = reference group. Statistical significance was based on a 95% CI that did not span 1.00. Bold values indicate significant results.

**Table 7:** Results for distal vaccination outcome analysis.

Variable	Trusters		Noncommitters		Skeptics		Overall Test		Noncommitters vs. Trusters		Skeptics vs. Trusters		Skeptics vs. Noncommitters	
	N = 106		N = 65		N = 32									
	Prob	SE	Prob	SE	Prob	SE	χ^2^	*p*	χ^2^	*p*	χ^2^	*p*	χ^2^	*p*
Vaccination refusal							**50.311**	**0** **.000**	0.012	0.913	**38.016**	**0** **.000**	**39.043**	**0** **.000**
Yes	0.357	0.061	0.347	0.068	0.922	0.055								
No or not sure	0.643	0.061	0.653	0.068	0.078	0.055								
COVID-19 vaccination							**29.095**	**0** **.000**	**10.74**	**0** **.001**	**23.33**	**0** **.000**	3.182	0.074
Yes	0.779	0.046	0.503	0.070	0.291	0.089								
No or not sure	0.221	0.046	0.497	0.070	0.709	0.089								
Annual influenza vaccination							**14.905**	**0** **.001**	3.323	0.068	**14.683**	**0** **.000**	2.913	0.088
Yes	0.384	0.050	0.233	0.061	0.075	0.063								
No or not sure	0.616	0.050	0.767	0.061	0.925	0.063								
COVID-19 vaccination intention							**142.07**	**0** **.000**	**8.684**	**0** **.034**	**114.342**	**0** **.000**	**27.533**	**0** **.000**
Yes, as soon as possible	0.327	0.053	0.173	0.049	0.000	0.000								
Yes, but wait and see	0.201	0.047	0.103	0.069	0.000	0.000								
Not sure	0.216	0.043	0.327	0.067	0.149	0.087								
No	0.256	0.047	0.397	0.066	0.851	0.087								
Annual influenza vaccination intention							**68.775**	**0** **.000**	**26.465**	**0** **.000**	**63.158**	**0** **.000**	**8.664**	**0** **.034**
Yes, as soon as possible	0.399	0.058	0.061	0.031	0.000	0.000								
Yes, but wait and see	0.187	0.042	0.255	0.058	0.147	0.076								
Not sure	0.133	0.037	0.246	0.057	0.122	0.071								
No	0.281	0.049	0.438	0.066	0.731	0.101								

Table notes. For the five categorical outcome variables, the DCAT command in Mplus returned probabilities (prob) and standard errors (SE). For the vaccination intention variables, the overall test had 6 degrees of freedom (df) and each pairwise comparison had 3 df. For the remaining variables, the overall test had 2 df and each pairwise comparison had 1 df. Bold values indicate statistically significant results with an alpha level of 0.05.

## Data Availability

The original contributions presented in this study are included in the article. Further inquiries can be directed to the corresponding author.

## Abbreviations

The following abbreviations are used in this manuscript:

LPA	Latent Profile Analysis
